# Global perspectives on pediatric pain management: analysis of attitudes, beliefs and knowledge in healthcare professionals, a multicenter study

**DOI:** 10.3389/fped.2026.1818534

**Published:** 2026-04-23

**Authors:** Inmaculada García-Valdivieso, Gustavo Correa de Amorim, Sonsoles Hernández-Iglesias, Mariana Alina Renghea, Pablo Pando Cerra, Auristela Chávez Vidalón de Mori, Esengül Elibol, Ana Frias, Sagrario Gómez-Cantarino

**Affiliations:** 1 Faculty of Physiotherapy and Nursing, University of Castilla-La Mancha, Toledo, Spain; 2 Department of Nursing in Hospital Care, Federal University of Triângulo Mineiro, Uberaba, Brazil; 3 Faculty of Health Sciences, Francisco of Vitoria University, Madrid, Spain; 4 DCIF University of Oviedo, Asturias, Spain; 5 Academic Department of Integrated Health Systems, National University of Ucayali, Pucallpa, Peru; 6 Nursing Department, Faculty of Health Sciences, Istanbul Bilgi University, Istanbul, Türkiye; 7 Comprehensive Health Research Center (CHRC), S. João de Deus School of Nursing, University of Évora, Évora, Portugal

**Keywords:** attitudes, beliefs, health professionals, non-pharmacological interventions, nursing education, pain management, pediatric care, pediatric pain

## Abstract

**Introduction:**

Pediatric pain remains insufficiently assessed and managed worldwide, despite advances in pain science and clinical guidelines. Health professionals' attitudes, beliefs, and level of training play a crucial role in the quality of pain management provided to children.

**Objectives:**

To analyze the relationship between attitudes and beliefs toward pediatric pain and the level of knowledge and training among health sciences students and professionals participating in an international educational project.

**Methods:**

A multicenter, cross-sectional, analytical study was conducted within the framework of the HUPEDCARE project. A total of 2,807 participants from Europe, America, Africa, Asia, and Oceania completed the validated HUPEDCARE-Q questionnaire. The instrument assessed attitudes, beliefs, knowledge and training, and clinical practices related to pediatric pain. Descriptive statistics, Pearson correlations, and multiple linear regression analyses were performed.

**Results:**

Participants showed moderate levels of knowledge regarding pediatric pain management (mean = 3.55 ± 0.73). Misconceptions persisted, particularly regarding opioid use and the interpretation of physiological indicators of pain. A significant positive correlation was found between attitudes and knowledge (*r* = 0.060; *p* = 0.001). Regression analysis showed that recognizing pain as a biopsychosocial experience and valuing non-pharmacological interventions were significant predictors of higher knowledge levels.

**Discussion:**

Despite increased awareness of pediatric pain as a multidimensional experience, relevant gaps in education and persistent misconceptions remain. Educational strategies addressing attitudes, beliefs, and clinical competencies are urgently needed to improve pediatric pain management and promote humanized care.

## 1 Introduction

Pain constitutes a multidimensional experience that significantly affects the physical, psychological, and social well-being of pediatric patients. Its subjective nature implies that each child perceives and interprets pain differently, which complicates its assessment through a universal standard instrument and represents a challenge for healthcare professionals in the implementation of effective strategies for the management and relief of pediatric pain (1, 2). Despite advances in healthcare, pediatric patients continue to experience pain associated with hospitalization, surgical interventions, and invasive procedures. This reality highlights that awareness and recognition of pain in the pediatric population among healthcare professionals have not yet reached an adequate level (3, 4).

Inadequate pain management in pediatric patients represents a problem of both clinical and ethical relevance. Scientific evidence demonstrates that untreated pain can lead to adverse short and long term consequences, negatively affecting child development (5, 6). The lack of effective pain relief raises significant ethical dilemmas, therefore, it is imperative that healthcare professionals adopt a patient centered care approach grounded in the humanization of care and in respect for the rights and well-being of children and their families (7, 8).

Nursing, as a fundamental component of the healthcare system, maintains continuous contact with patients and occupies a privileged position to observe, assess, and intervene in the management of pediatric pain. Within the multidisciplinary team, nursing plays an essential role in pain management through the implementation of pharmacological and non-pharmacological interventions aimed at relieving pediatric pain (9, 10).

However, to ensure an optimal, evidence based approach, it is essential that healthcare teams possess up to date knowledge, clinical skills, and appropriate attitudes that enable them to assess and manage pediatric pain in a comprehensive manner (11).

Pain management is a complex process that requires the integration of multiples strategies and therapeutic methods aimed at mitigating or eliminating suffering. Beyond clinical skills, effective pain management demands ethical sensitivity and a commitment to the protection and care of pediatric patients (12, 13). However, studies indicate that healthcare professional's attitudes, beliefs, and knowledge regarding pain are often insufficient or affected by cognitive biases, which limits the effectiveness and quality of the care provided (14, 15).

The interdisciplinary team working with pediatric patients faces multiple challenges arising from factors such as young children's limited ability to verbally communicate pain, deficits in knowledge and the presence of misconceptions related to pain management, difficulties in selecting validated and age-appropriate pain assessment instruments for the pediatric population, as well as the lack of standardized protocols and institutional guidelines to support clinical practice (16, 17).

According to the American Pain Society, pain was recognized as the fifth vital sign with the aims of preventing its underestimation and promoting appropriate management (18). However, clinical practice continues to reveal significant deficiencies in pain assessment and control (19). During and after hospitalization, pediatric patients may experience pain as a result of various invasive interventions performed for diagnostic and therapeutic purposes, such as venipuncture, intravenous catheterization, or the administration of injections, underscoring the importance of the role of healthcare professionals in its prevention and relief (20).

Several studies have shown that deficiencies in this knowledge among healthcare teams constitute one of the most limiting factors for effective pediatric pain management (21–23). Consequently, strengthening competencies and knowledge related to pain assessment and treatment is considered a priority in order to optimize the quality of healthcare delivery (24). The multidisciplinary team providing care to pediatric patients experiencing pain can play a leadership role throughout the entire care process, including the child's psychological and emotional preparation, accompaniment and support during procedures, pain assessment, and the selection of therapeutic interventions aimed at pain relief (25).

The development of these professional competencies begins in the university setting, where higher education occupies a strategic position in reducing the existing knowledge gap in pediatric pain management by providing opportunities that facilitate the acquisition and application of scientific and clinical skills among future generations of healthcare professionals (26). The implementation of comprehensive educational programs encompassing both theoretical and practical components related to pathophysiology, pain assessment, monitoring, and therapeutic interventions will contribute to strengthening professional training and specialization in this area (27).

Nevertheless, uncertainty persists as to whether the content related to pediatric pain management included in Health Sciences studies is sufficient to ensure the training of professionals competent in the effective assessment and management of pain (28). The literature suggests that such educational programs often present limitations in the incorporation of specific and up to date content, thereby hindering adequate preparation to meet the needs of pediatrics patients (29, 30).

However, evidence demonstrates that specific training programs focused on pain management significantly improve healthcare professional's attitudes, beliefs, and clinical competencies, promoting evidence based practice oriented toward child well-being. To ensure optimal care, Health Sciences students must possess and adequate level of knowledge about pain, as well as appropriate attitudes toward its assessment and clinical management (31, 32).

The relationship between healthcare professional's beliefs about pain and their attitudes toward its treatment remains an underexplored area. In order to design and implement effective educational programs, it is essential to identify and evaluate the level of knowledge, attitudes, and beliefs regarding pediatric pain held by both future and current healthcare professionals. Understanding these factors will enable the development of educational strategies that promote more humane, ethical, and evidence based practices (33, 34).

For this reason, an international research initiative entitled “Higher Education as a Driver in the Humanisation of Pediatric Pain Care (HUPEDCARE)” has been promoted and developed, funded by the European Union (EU). This transnational cooperation project involves 15 universities from three continents (Europe, America and Africa), which are listed in Table 1.

**Table 1:** Universities participating in the HUPEDCARE project.

Universities by continent	Countries	Names of Universities
Universities of Europe	Spain	University of Castilla-La Mancha
		Francisco of Vitoria University (Madrid)
		University of Oviedo (Asturias)
	Portugal	Santarém Polytechnic Institute
		University of Évora
	Poland	Medical University of Gdansk
	Turkey	Istanbul Bilgi University
Universities of Africa	Cape Verde	University of Cape Verde
		Jean Piaget University of Cape Verde
	Mozambique	Licungo University of Mozambique
		Eduardo Mondlane University of Mozambique
Universities of America	Brazil	Federal University of São Carlos
		University of São Paulo
	Peru	National University of Trujillo
		National University of Ucayali

Within this context, the present study aims to analyze the association between attitudes and beliefs and the knowledge and training of healthcare professionals involved in the management and relief of pediatric pain, with the purpose of contributing to the improvement of the quality of pediatric care. Based on the result obtained, this study seeks to expand the body of disciplinary knowledge in Health Sciences, strengthen child centered care, and promote ethical and scientific approaches in pediatric healthcare.

## 2 Methods

A quantitative, cross-sectional, analytical, and correlational study was conducted to examine whether attitudes and beliefs are associated with the level of knowledge and training of current and future health professionals regarding pediatric pain management, as well as their relationship with sociodemographic variables such as sex, age, profession, and country of origin. This study followed the guidelines of the STROBE statement for cross-sectional studies (35). The research was carried out within the framework of the international HUPEDCARE project. As a multicenter study, the sample consisted of a total of 2,807 participants from multiple countries across the five continents: Europe, America, Africa, Asia and Oceania.

Participants included higher education students, healthcare professionals, and faculty members and researchers affiliated with Health Sciences programs, all aged over 18 years and having provided written informed consent. Data were collected between February 1 and August 14, 2025, and incomplete questionnaires were excluded. A non-probabilistic, purposive sampling method based on voluntary participation was employed. Data were collected using the “Humanisation of Pediatric Care in Pain Management with a Non-Pharmacological Approach” questionnaire (HUPEDCARE-Q), which was designed and validated by the research team (36).

The instrument included and introductory information section comprising a set of questions designed to determine the participant's sociodemographic and professional characteristics, such as sex, age, country of origin, work setting, profession, and/or field of study. The questionnaire consisted of 28 items, distributed across three dimensions: attitudes and beliefs about pediatric pain (11 items), knowledge and training pain management (7 items), and good practices in pediatric pain management (10 items). The first two dimensions were assessed using a 5-point Likert-type scale, ranging from 0 (strongly disagree) to 5 (strongly agree). Cumulative higher scores served as a proxy for enhanced clinical competence in pediatric pain assessment and management, reflecting both a robust theoretical framework and the systematic integration of practical training and validated tools (37–41). In this study, the analysis focused exclusively on the dimensions of attitudes and beliefs and knowledge and training, as both share a homogeneous response structure (a 0–5 Likert scale), which allows for coherent data aggregation to evaluate theoretical and attitudinal competence. The third dimension good practices, was excluded from the overall quantitative analysis to avoid variability bias, thereby ensuring greater robustness and internal consistency in the calculation of the reported clinical competence.

The score range obtained across the two analyzed dimensions ranged from a minimum of 0–18 points to a maximum of 90 points. This upper limit derives from the sum of both scales: attitudes and beliefs, consisting of 11 items with a maximum possible score of 55 points, and knowledge and training, consisting of 7 items with a maximum possible score of 35 points. In this study, a combined score of less than 45 points across both dimensions was considered indicative of a negative impact, reflecting greater deficiencies in attitudes, beliefs, knowledge, training, and good practices related to pediatric pain management. In contrast, a higher score indicates better performance in the assessment and treatment of pediatric pain, as well as greater integration of practical training and the use of appropriate tools (36).

The psychometric reliability of the questionnaire was evaluated through an internal consistency analysis. Cronbach's alpha coefficients obtained were 0.86 for the attitudes and beliefs dimension, and 0.80 for the knowledge and training dimension (36). According to established psychometric standards for clinical research, these values demonstrate satisfactory reliability and adequate item homogeneity, ensuring that the instrument consistently measures the intended underlying constructs (42). The sampling adequacy analysis yielded a Kaiser-Meyer-Olkin (KMO) index of 0.90, indicating good suitability for Exploratory Factor Analysis (EFA). Bartlett's test of sphericity was significant [*χ*^2^ (153) = 6,650.48, *p* < 0.001], confirming the appropriateness of the data. The factor analysis extracted two factors with eigenvalues greater than 1, which explained 57.3% of the total variance (36).

The study variables included age, sex, country of origin, profession, work setting, attitudes and beliefs about pain, knowledge and training, and clinical best practices. The generated scores were treated as continuous quantitative variables. Data were analyzed using IBM SPSS software, version 24. Descriptive statistics were performed, including means, standard deviations, frequencies, and percentages. Analysis of variance (ANOVA) was used to compare mean scores across subgroups. Multiple linear regression models were applied to identify predictors of attitudes, beliefs, knowledge, and training, while controlling for the effects of sociodemographic variables. The level of statistical significance was set at *p* < 0.05.

The questionnaire was administered online and anonymously through the HUPEDCARE platform, a secure system managed by the participating universities. By specifically targeting health sciences students and healthcare professionals, the study leveraged a sample with a standardized academic and scientific background. This shared educational framework acts as a cohesive factor that mitigates sociocultural variability, as participants possess a common technical lexicon and similar clinical training on a global scale. Furthermore, the HUPEDCARE platform was engineered to automatically present the questionnaire in the official language of the participant's respective country or region. All instruments underwent a rigorous process of translation and cross-cultural adaptation, ensuring that questions were tailored to local linguistic nuances while maintaining strict conceptual equivalence across all versions.

The study received approval from the Social Research Ethics Committee (CEIS) of the University of Castilla-La Mancha, as the coordinating institution of the project, under registration number CEIS-2025-91937, on January 30, 2025. The informed consent form explained the purpose and scope of the research. Anonymity and data confidentiality were guaranteed, and the data were used exclusively for scientific purposes. Participation in the study was entirely voluntary, and participants were free to withdraw at any time.

## 3 Results

The sample of the multicenter international study consisted of 2,807 participants from the five continents: Europe (*n* = 1,695; 60.4%), America (*n* = 687; 24.5%), Africa (*n* = 405; 14.4%), Asia (*n* = 14; 0.5%), and Oceania (*n* = 6; 0.2%). A clear predominance of female participants was observed (80.5%). The 21–30-year age group was the most represented (58.1%), consisting mainly of Health Sciences students (61.6%), followed by healthcare professionals (29.1%). Nursing was the most represented healthcare speciality (72.2%), followed by Medicine (20.3%), as shown in Table 2.

**Table 2:** Sociodemographic data of the sample (*n* = 2,807).

Variable	Europe	America	Africa	Asia	Oceania	Total
Sex	*n* (%)					
Female	1,411 (83.3)	517 (75.3)	324 (80.0)	6 (42.9)	3 (50.0)	2,261 (80.5)
Male	282 (16.6)	170 (24.7)	80 (19.8)	8 (57.1)	3 (50.0)	543 (19.3)
Another	2 (0.1)	—	1 (0.2)	—	—	3 (0.1)
Age						
Under 20 years	201 (11.9)	13 (1.9)	8 (2.0)	—	—	222 (7.9)
21–30 years	1,021 (60.2)	422 (61.4)	184 (45.4)	3 (21.4)	—	1,630 (58.1)
31–40 years	174 (10.3)	111 (16.2)	99 (24.4)	—	—	384 (13.7)
41–50 years	138 (8.1)	64 (9.3)	82 (20.2)	8 (57.1)	3 (50.0)	295 (10.5)
>50 years	161 (9.5)	77 (11.2)	32 (7.9)	3 (21.4)	3 (50.0)	276 (9.8)
Work setting						
Healthcare	367 (21.7)	230 (33.5)	212 (52.3)	4 (28.6)	3 (50.0)	816 (29.1)
Teacher/Researcher	147 (8.7)	61 (8.9)	42 (10.4)	8 (57.1)	3 (50.0)	261 (9.3)
Student	1,181 (69.7)	396 (57.6)	151 (37.3)	2 (14.3)	—	1,730 (61.6)
Professional Area						
Nursing	1,458 (86.0)	396 (57.6)	167 (41.2)	4 (28.6)	1 (16.7)	2,026 (72.2)
Medicine	119 (7.0)	218 (31.7)	224 (55.3)	7 (50.0)	2 (33.3)	570 (20.3)
Physiotherapy	61 (3.6)	49 (7.1)	1 (0.2)	—	—	111 (4.0)
Nursing Assistant	14 (0.8)	1 (0.1)	—	—	—	15 (0.5)
Others	43 (2.5)	23 (3.3)	13 (3.2)	3 (21.4)	3 (50.0)	85 (3.0)

The analysis of the mean scores of the items from the dimensions “attitudes and beliefs about pediatric pain” and “knowledge and training in pediatric pain” is presented in Table 3. To calculate the overall mean scores for each dimension, the values of the following negatively worded items were reverse-coded: item C1 from the knowledge and training dimension, and items A1, A2, A3, A8, A9, A10, and A11 from the attitudes and beliefs dimension.

**Table 3:** Descriptive statistics of the items of the “attitudes and beliefs in pediatric pain” and “knowledge and training” scales.

Scale—Attitudes and Beliefs in Pediatric Pain	Mean (SD)
A1—Because the neurological system is still developing in children under 2 years of age, they have reduced sensitivity to pain and diminished memory of painful experiences.	2.87 (1.44)
A2—Similar stimuli produce the same intensity of pain in different children.	2.42 (1.38)
A3—Children under 6 months of age cannot tolerate opioids for pain relief.	3.37 (1.35)
A4—After the recommended initial dose of analgesics, subsequent doses should be individualized according to the patient's response.	4.48 (1.35)
A5—The use of non-pharmacological pain interventions is recommended as a standalone approach rather than in combination with analgesic medications.	3.32 (1.49)
A6—Pediatric pain is a personal experience influenced by biological, psychological, and social factors.	4.49 (1.39)
A7—Non-pharmacological interventions (breastfeeding, kangaroo mother care, oral sucrose or glucose, and non-nutritive sucking) are highly effective for the management of mild to moderate pain, but are rarely useful for more severe pain.	3.96 (1.26)
A8—Parents should not be present during painful procedures.	2.56 (1.45)
A9—Children experiencing pain should be encouraged to endure it as much as possible before resorting to pain relief measures.	2.15 (1.31)
A10—Administering placebos (e.g., sterile water or saline solution) to children is often a useful test to determine whether pain is real.	3.28 (1.40)
A11—The use of opioids for the treatment of acute pain may lead to addiction in pediatric patients.	3.71 (1.37)
**Mean total score**	**4**.**12** **(0**.**73)**
**Scale—Knowledge and Training in Pediatric Pain**	
C1—To confirm the presence of severe pain in a child, assessment should be based on the observation of changes in vital signs.	3.92 (1.40)
C2—I am familiar with and apply pain assessment scales in children.	3.97 (1.43)
C3—I am familiar with and apply the WHO linear analgesic ladder for pain management in children.	3.77 (1.34)
C4—Training on acute pain in children and its management is sufficient.	3.07 (1.25)
C5—I am able to identify early signs of pain in newborns.	3.49 (1.34)
C6—I know how to manage acute pain in children.	3.60 (1.28)
C7—Analgesia should be administered prior to performing traumatic diagnostic procedures.	3.90 (1.37)
**Mean total score**	**3**.**55** **(0**.**73)**

The weakest point in the knowledge and training dimension was self-perceived training. Health Sciences professionals and students acknowledged that their training in acute pediatric pain and its management was sufficient only to a limited extent (mean = 3.07), indicating that professionals recognize having received inadequate training in this area. A common technical misconception was also identified, reflected by a high mean score (3.92) on item C1, which states that the confirmation of severe pain in a child should be based on the observation of changes in vital signs. This finding suggests that many professionals still erroneously believe that if heart rate or respiratory rate does not increase, the child is not experiencing pain.

Regarding the attitudes and beliefs dimension, among the negatively worded items, rejection of opioid use stood out (mean = 3.71). Both professionals and students showed high mean scores on this item, indicating that many still believe opioids may cause addiction in children, which can lead to under treatment of pain. Notably, among the positively worded items, a high mean score (4.49) was observed for the recognition that pain in childhood is a subjective experience influenced by biological, psychological, and social factors.

The study hypothesis posits that attitudes and beliefs aligned with scientific evidence regarding pediatric pain are positively and significantly associated with the level of theoretical knowledge and practical training among both health sciences students and professionals. Furthermore, it is postulated that these attitudinal factors serve as significant predictors of clinical competency in pediatric pain management.

Pearson's correlation analysis demonstrated a positive and statistically significant, although weak, association between the mean score of attitudes and beliefs and the mean score of knowledge and training in pediatric pain (*r* = 0.060; *p* = 0.001) (Table 4). This finding suggests that attitudes more closely aligned with scientific evidence are associated with greater theoretical knowledge.

**Table 4:** Pearson's correlation between “attitudes and beliefs” and “knowledge and training in pediatric pain”.

Correlation between variables		Total positive attitude index based on the scale of attitudes and beliefs regarding paediatric pain	Average score for knowledge and training in pediatric pain
Variable 1			
Total positive attitude index based on the scale of attitudes and beliefs regarding paediatric pain.	Pearson Correlation	1	0.060
	Sig. (2-tailed)		0.001
	*N*	2,807	2,807
Variable 2			
Average score for knowledge and training in pediatric pain.	Pearson Correlation	0.060	1
	Sig. (2-tailed)	0.001	
	*N*	2,807	2,807

Correlation is significant at the 0.01 level (2-tailed).

To test the influence hypothesis, a multiple linear regression analysis was conducted, as shown in Table 5. The final model explained approximately 6.9% of the variance in knowledge of pediatric pain (adjusted *R*^2^ = 0.065; *F* (11.2795) = 18.69; *p* < 0.001).

**Table 5:** Multiple linear regression model: association between attitudes and beliefs and knowledge and training scores in pediatric pain.

Variable	Coefficient *B* (Standard Deviation)	(*β*)*	*p* value
Constant	2.889 (0.113)	—	**<0**.**001**
A1	0.012 (0.011)	0.23	0.272
A2	−0.061 (0.010)	−0.115	**<0**.**001**
A3	0.045 (0.011)	0.083	**<0**.**001**
A4	0.031 (0.011)	0.057	**0**.**006**
A5	0.014 (0.010)	0.029	0.144
A6	0.050 (0.011)	0.094	**<0**.**001**
A7	0.090 (0.011)	0.155	**<0**.**001**
A8	−0.008 (0.010)	−0.016	0.417
A9	−0.026 (0.012)	−0.046	**0**.**029**
A10	0.018 (0.011)	0.034	0.092
A11	0.019 (0.011)	0.036	0.074

*B* = Non-standardised coefficient; β* = Standardised coefficient (indicates the strength of the influence).

Statistically significant values are indicated in bold.

The attitudes and beliefs that acted as significant positive predictors of a higher level of knowledge and training were: recognizing that “pain in childhood is a personal experience influenced by biological, psychological, and social factors” (*β* = 0.094; *p* < 0.001), and “affirming that non-pharmacological interventions are effective for the control of mild to moderate pain” (*β* = 0.115; *p* < 0.001).

Conversely, misconceptions were identified that acted as barriers, such as the belief that “similar stimuli in different children produce the same intensity of pain” and that “children in pain should be encouraged to endure pain as much as possible before resorting to pain relief measures”. These beliefs functioned as significant negative predictors (*β* = −0.115; *p* < 0.001 and *β* = −0.046; *p* = 0.029, respectively), indicating that professionals who disregard the subjective nature of pain tend to have lower levels of technical knowledge regarding its assessment and management.

## 4 Discussion

The present multicenter and international study provides relevant evidence on the relationship between attitudes, beliefs, and levels of knowledge and training in the management of pediatric pain among students and healthcare professionals in Health Sciences. The results confirm that, although there is a general recognition of the multidimensional nature of childhood pain, misconceptions and educational gaps persist, continuing to hinder optimal pain assessment and management across different educational and clinical settings, as previously described in earlier research (21–23, 43–45).

One of the most relevant findings is the insufficient self-perceived training in pediatric pain, reflected in the low score of the item related to the adequacy of education on acute childhood pain. This finding aligns with a sustained body of evidence highlighting chronic structural deficits in Health Sciences studies. As early as 2018, research underscored the lack of standardized education in this field (46), a concern that remains critically relevant according to recent studies conducted between 2020 and 2024. These contemporary findings confirm that pediatric pain education continues to be limited, fragmented, or outdated across various international contexts (27, 28). Such persistent educational gaps hinder the acquisition of robust clinical competencies, suggesting that despite years of academic warnings, the integration of evidence-based pain management into formal health studies has not yet achieved the necessary depth to ensure high-quality pediatric care (47).

Furthermore, our analysis identified a highly prevalent technical error of considerable clinical relevance: the persistent belief that the assessment of severe pain should be primarily based on fluctuations in vital signs. This finding reinforces previous evidence (5, 48, 49), warning that excessive reliance on physiological indicators, such as tachycardia or hypertension, frequently leads to systematic underestimation and undertreatment of pain, particularly in neonates and preverbal children. From a pathophysiological perspective, hemodynamic responses lack specificity, as they may be influenced by stress, agitation, or metabolic instability. Moreover, they exhibit a rapid habituation phenomenon, often returning to baseline even while the painful stimulus persists. The prevalence of this misconception among participants suggests a significant gap in understanding the dissociation between physiological stress and the subjective experience of pain in a developing nervous system. Consequently, our findings underscore the urgent need to move from monitoring based solely on vital signs toward the implementation of validated, multidimensional pain scales adapted to developmental maturity (50). Such tools are essential to capture behavioral and contextual nuances that physiological data alone fail to reflect, thereby ensuring safer and more ethically sound pediatric practice.

With regard to attitudes and beliefs, the results reveal a coexistence of approaches aligned with scientific evidence and persistent misconceptions. On the one hand, a high level of agreement was observed with the statement that pediatric pain is a personal experience influenced by biological, psychological, and social factors, reflecting a progressive incorporation of the biopsychosocial model of pain. This approach has been widely supported in the literature as a fundamental pillar for comprehensive and humanized pediatric pain management (2, 7, 8, 51, 52).

However, in parallel, a significant fear regarding the use of opioids in the pediatric population was observed, associated with the belief of a high risk of addiction. This phenomenon has been reported both among students and healthcare professionals and is associated with conservative and insufficient analgesic practices (43, 45, 47). The persistence of this attitude suggests that the safe use of opioids in pediatrics continues to represent a barrier to adequate pain control. Nevertheless, the available evidence indicates that this fear, although understandable, is not always supported by robust clinical data when opioids are used appropriately (53). Strengthening the appropriate use of opioids in pediatric care requires not only evidence based clinical guidelines but also educational strategies aimed at healthcare professionals both in training and in clinical practice (54). In fact, a narrative review advocates for greater precision in the individual assessment of opioid requirements, together with knowledge of prevention and monitoring measures, stating that this approach may help reduce unjustified barriers and improve the quality of pain management in the pediatric population (55).

Correlational analysis revealed a positive, albeit weak, association between attitudes and beliefs and the level of knowledge and training, indicating that attitudes more closely aligned with scientific evidence tend to coexist with higher levels of theoretical knowledge. This finding is consistent with educational models suggesting that knowledge alone is insufficient to modify clinical practice and must be accompanied by changes in professional beliefs and values (56, 57). The weakness of this relationship suggests the presence of invisible barriers, such as deeply rooted hospital culture, the lack of clear protocols, or the inertia of previous clinical experience may exert an equal or even greater influence than academic training. Therefore, our study demonstrates that improving the management of pain in children cannot rely solely on increasing theoretical education; it also requires interventions targeting the institutional environment and the shared values that shape professionals' daily practice. Furthermore, international literature underscores that these attitudes vary across cultural contexts; while traditional attitudes prevail in Southeast Asia (58, 59), myths regarding higher pain tolerance in childhood persist in Oceania (60). These global divergences reinforce the imperative need to implement standardized clinical protocols to minimize subjectivity and ensure equitable and safe care.

The results of the regression model reinforce this interpretation by identifying as positive predictors of knowledge and training those beliefs that acknowledge the subjective and individual nature of pediatric pain, as well as confidence in the effectiveness of non-pharmacological interventions for mild to moderate pain. These findings are consistent with the literature highlighting the value of non-pharmacological strategies, particularly when they are integrated as a complement to pharmacological analgesia and when the active involvement of parents and caregivers is promoted (61–65).

In contrast, beliefs such as assuming that the experience of pain is uniform across children or that pain should be tolerated before intervention have been identified as negative predictors, constituting a barrier to the development of adequate clinical competencies in pediatric pain management (66, 67). These results suggest that the persistence of approaches derived from traditional models continues to limit the implementation of appropriate analgesic practices (55). From an ethical perspective, such beliefs have been questioned for contradicting the principles of patient centered care and the humanization of care (2, 7, 8).

Overall, the results of this study support the need for specific, cross-cutting, and culturally adapted educational interventions, both in higher education training and in the continuing professional development of healthcare professionals. In this regard, the HUPEDCARE project emerges as a strategic initiative by addressing pediatric pain management from a global, interdisciplinary, and care humanization centered perspective, aligning with international recommendations aimed at improving the quality and equity of pediatric pain care (1, 2).

## 5 Limitations

This study has several limitations that should be considered when interpreting the findings. The sample may not be fully representative of the overall population of students and healthcare professionals in the health sciences worldwide, particularly in regions with lower participation or underrepresentation, such as Asia and Oceania. In addition, relevant factors such as specific clinical experience, continuing education, institutional culture, and the availability of protocols and resources may significantly influence outcomes and should be explored in future research.

## 6 Conclusions

This multicenter, international study confirms that, although there is general recognition of the multidimensional nature of pediatric pain, misconceptions and educational gaps persist that limit optimal pain assessment and management among current and future health sciences professionals. Insufficient self-perceived training, together with technical shortcomings such as the overreliance on physiological indicators to assess severe pain, underscores the need to strengthen evidence based education and the systematic use of validated pain assessment tools tailored to the child's age and developmental stage.

Correlational and regression analyses indicate that evidence aligned attitudes and beliefs are associated with greater theoretical knowledge, however, contextual factors such as clinical experience, institutional culture, and the availability of protocols also exert a significant influence on pediatric pain management practices. Therefore, overcoming cognitive and attitudinal barriers requires comprehensive interventions that integrate education, practical training, and the promotion of care humanization, as well as further research to elucidate the role of these contextual factors and to advance comprehensive, ethical, and evidence based pediatric pain care.

## Data Availability

The original contributions presented in the study are included in the article/Supplementary Material, further inquiries can be directed to the corresponding author.
